# Comparison of droplet digital PCR and conventional quantitative PCR for measuring *EGFR* gene mutation

**DOI:** 10.3892/etm.2015.2221

**Published:** 2015-01-27

**Authors:** BO ZHANG, CHUN-WEI XU, YUN SHAO, HUAI-TAO WANG, YONG-FANG WU, YE-YING SONG, XIAO-BING LI, ZHE ZHANG, WEN-JING WANG, LI-QIONG LI, CONG-LI CAI

**Affiliations:** 1 Department of Pathology, Affiliated Hospital of Academy of Military Medical Sciences, Beijing 100071, P.R. China; 2 Wuhan YZY Medical Science and Technology Co., Ltd., Wuhan, Hubei 430075, P.R. China

**Keywords:** epidermal growth factor receptor gene mutation, non-small cell lung cancer, droplet digital polymerase chain reaction, quantitative polymerase chain reaction

## Abstract

Early detection of epidermal growth factor receptor (*EGFR*) mutation, particularly *EGFR* T790M mutation, is of clinical significance. The aim of the present study was to compare the performances of amplification refractory mutation system-based quantitative polymerase chain reaction (ARMS-qPCR) and droplet digital polymerase chain reaction (ddPCR) approaches in the detection of *EGFR* mutation and explore the feasibility of using ddPCR in the detection of samples with low mutation rates. *EGFR* gene mutations in plasmid samples with different T790M mutation rates (0.1–5%) and 10 clinical samples were detected using the ARMS-qPCR and ddPCR approaches. The results demonstrated that the ARMS-qPCR method stably detected the plasmid samples (6,000 copies) with 5 and 1% mutation rates, while the ddPCR approach reliably detected those with 5% (398 copies), 1% (57 copies), 0.5% (24 copies) and 0.1% (average 6 copies) mutation rates. For the 10 clinical samples, the results for nine samples by the ARMS-qPCR and ddPCR methods were consistent; however, the sample N006, indicated to be *EGFR* wild-type by ARMS-qPCR, was revealed to have a clear *EGFR* T790M mutation with seven copies of mutant alleles in a background of 6,000 wild-type copies using ddPCR technology. This study demonstrates the feasibility of applying the ddPCR system to detect *EGFR* mutation and identified the advantage of ddPCR in the detection of samples with a low *EGFR* mutation abundance, particularly the secondary *EGFR* T790M resistance mutation, which enables early diagnosis before acquired resistance to tyrosine kinase inhibitors becomes clinically detectable.

## Introduction

Lung cancer is the leading cause of cancer mortality and non-small cell lung cancer (NSCLC) accounts for ~80% of lung cancer cases (1). Epidermal growth factor receptor (*EGFR*) is frequently overexpressed in NSCLC and it is a promising target for individualized therapy (2). *EGFR*-targeted therapy with small-molecule tyrosine kinase inhibitors (TKIs), including gefitinib and erlotinib, has been approved for the treatment of advanced NSCLC (3). These inhibitors function by competitively binding at the adenosine triphosphate (ATP)-binding cleft of the receptor kinase domain, thereby blocking kinase activation and subsequent downstream signal transduction (3). However, TKIs are not effective in all NSCLC patients; an activating *EGFR* mutation is a precondition for sensitivity (1).

Among the 29 types of common *EGFR* gene mutation, small in-frame deletions in exon 19 and heterozygous mutations of exon 21 (most commonly producing the L858R substitution), which is around the ATP-binding pocket, are the mutation hotspot, constituting 85–90% of all *EGFR* mutations (3,4). The correlation between *EGFR* mutations and *EGFR* TKI sensitivity has been validated in several clinical trials and has been demonstrated to have potential prognostic value (5–7). Nevertheless, the majority of tumors become resistant to TKIs, mostly due to the incidence of a secondary T790M mutation, which is reported to negate the hypersensitivity of activating *EGFR* mutations (8).

Numerous methods for detecting *EGFR* mutation and thereby predicting the response to *EGFR*-targeting inhibitor therapy have emerged. Fluorescence *in situ* hybridization (FISH) is a strong predictor of survival benefit in patients with advanced NSCLC treated with *EGFR* inhibitors; however, it is labor intensive (9). Standard DNA-sequencing is able to detect tumor mutations within an abundance of 10–25%, depending on the quality of the tumors and genomic materials (10). The Scorpion™ amplification-refractory mutation system (ARMS™) technique has a detection limit of ~1% (11). Methods based on quantitative polymerase chain reaction (qPCR) have been developed to evaluate mutation of the *EGFR* gene and are widely used clinically.

Recently, a new platform, named droplet digital polymerase chain reaction (ddPCR), has developed and is widely used for many clinical applications due to its unparalleled sensitivity and precision. ddPCR can quantify mutations in the *EGFR* gene in lung cancer at the single-molecule level, making it possible to detect samples with low mutation rates (12,13). ddPCR uses a water-in-oil emulsion to generate up to 20,000 nanoliter-sized droplets that each contain no or some copies of a template and undergo separate end-point amplifications. A fluorescence detector is used to analyze these droplets, and a Poisson modeling equation is applied to measure the absolute number of copies of the target sequences.

In the present study, ARMS-qPCR and ddPCR methods were used to analyze plasmid samples and clinical samples, with the aim of comparing the performances of the two methods and exploring the feasibility of using ddPCR in the detection of samples with low mutation rates. The significance of and prospects for ddPCR in the detection of *EGFR* mutation, particularly the secondary *EGFR* T790M resistance mutation, were assessed. To the best of our knowledge, the present study is the first concerning the use of ddPCR for the detection of the *EGFR* T790M mutation and preliminarily illustrates the clinical significance of its early detection.

## Materials and methods

### Plasmid construction and plasmid sample preparation

Plasmid containing the *EGFR* T790M mutation was constructed as follows: Genomic DNA of HT-29 cells (ATCC^®^ HTB-38™; American Type Culture Collection, Manassas, VA, USA) was isolated using the TIANamp Blood DNA kit (cat. no. DP318-02; Tiangen Biotech Co., Ltd, Beijing, China). A DNA fragment containing the *EGFR* gene was amplified from the genomic DNA of HT-29 cells using specific primers (Table I), and the PCR product was then ligated to pEASY-T1 vector (cat. no. CT101; TransGen Biotech Co., Ltd., Beijing, China). DNA sequencing was used to verify the accuracy of the fragment. The Fast Mutagenesis System was used to construct the *EGFR* T790M mutation with specific primers (Table I) following the manufacturer’s instructions (cat. no. FM111; TransGen Biotech Co., Ltd.). The distinct T790M mutation was verified by sequencing. Different amounts of T790M mutant plasmids were mixed with wild-type *EGFR* plasmid to yield 6, 30, 60 and 300 copies of mutant molecules in 6,000 wild-type molecules, that is 0.1, 0.5, 1 and 5% mutation rates, respectively. Haploid copy number dilutions were calculated based on the molecular weight of one normal haploid female genome equalling 3.275 pg (12). Quantification was performed by the standard curve method using five standard dilutions, each in duplicate, of normal female genomic DNA ranging from 330 to 0.528 ng (100,000 to 160 haploid genome copies) per reaction.

### Formalin-fixed paraffin embedded (FFPE) tumor samples from NSCLC patients

FFPE tumor samples obtained from patients between 2013 and 2014 were collected from the Affilated Hospital of Academy of Military Medical Sciences (Beijing, China). Written informed consent was obtained from all of the patients. The present study procedures were approved by the Institutional Review Board of the Affiliated Hospital of Academy of Military Medical Sciences. All tumor samples were routinely assessed by sectioning, hematoxylin and eosin staining, and visualization under a microscope to ensure that the tumor content was ≥50%. If the tumor content was observed to be <50%, the samples were trimmed to satisfy the criteria. Genomic DNA was extracted using the QIAamp DNA FFPE Tissue kit (cat. no. 56404; Qiagen, Shanghai, China). Genomic DNA (gDNA) concentrations were determined by the measurement of optical density at 260 nm (OD260) using a Nanodrop 2000 spectrophotometer (Thermo Fisher Scientific, Wilmington, DE, USA) and the purity was evaluated by measurement of the OD260/OD280 ratio (all gDNA samples were between 1.92 and 1.97).

### qPCR

The *EGFR* gene mutation statuses of the plasmid samples and 10 FFPE tumor samples were assessed using the Human EGFR Gene Mutations Detection kit (YZYMT-002-C; Wuhan YZY Medical Science and Technology Co., Ltd., Wuhan, China), to detect the 29 common *EGFR* mutations (G719A, G719S, G719C, S768I, T790M, L858R, L861Q, V769-D770insASV, H773-V774insH, D770-N771insG and the 19 common short in-frame deletions previously reported for *EGFR* exon 19). The mutation assays were labeled with FAM, and each reaction mixture also contained a positive PCR control labeled with VIC. The templates were adjusted to a unified 10 ng/μl concentration, and the qualities of the templates were monitored and assessed by the reference system of the kit. qPCR was performed using a 7500 Real Time PCR system (Applied Biosystems Life Technologies, Shanghai, China). Thermocycling conditions were 95°C for 5 min, followed by 40 cycles of 95°C for 15 sec and 60°C for 1 min. No template control (NTC) reactions were performed using water with no template, and in all cases, no amplification occurred.

### ddPCR analysis

ddPCR was then performed to detect the *EGFR* gene mutation statuses of the plasmid samples and the 10 FFPE tumor samples using a QX200 Droplet Digital PCR system (Bio-Rad Laboratories, Inc., Hercules, CA, USA). Reactions were performed in 25 μl volumes using 12.5 μl ddPCR 2X Master mix (Bio-Rad Laboratories, Inc.), 1.25 μl 20X primer and TaqMan Probe mix (Applied Biosystems Life Technologies), 8.75 μl nuclease-free water and 2.5 μl template. Each sample was then loaded into the well of a droplet generator cartridge; 20 μl sample was transferred into the middle wells of the cartridge, being careful to avoid bubbles and 70 μl droplet generation oil (Bio-Rad Laboratories, Inc.) was added to the lower wells. The sample-containing cartridge was placed into the droplet generator to generate individual droplets. Once the process was complete, 40 μl droplets were transferred into the wells of a 96-well PCR plate, sealed, and loaded into the thermal cycler. The following program was run: 95°C for 10 min, followed by 40 cycles of 94°C for 30 sec and 58°C for 1 min, followed by 98°C for 10 min, and holding at 4°C. After PCR was complete, the sealed plate was loaded into the droplet reader for the detection of complete ddPCR reactions in individual droplets. The data was analyzed using QuantaSoft software (Bio-Rad Laboratories, Inc.) with the thresholds for detection set manually based on results from NTC wells containing water instead of DNA and the negative control using genomic DNA of HT-29 cells.

## Results

### EGFR mutation detection of the plasmid samples

**Figure 1:**
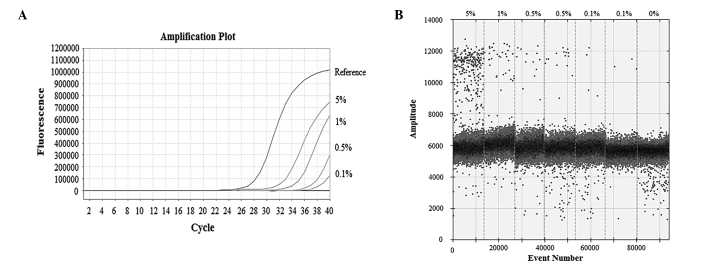
Quantitative performances of ARMS-qPCR and ddPCR for *EGFR* mutation analysis. *EGFR* mutation detection results of the plasmid samples with 5–0.1% mutation rates by (A) ARMS-qPCR and (B) ddPCR methods. Mixtures of wild-type *EGFR* and T790M mutant plasmids present at fractional concentrations of 0.1 to 5% were analyzed with ARMS-qPCR and ddPCR. The total number of *EGFR* sequences in the sample was 6,000 per sample. EGFR, epidermal growth factor receptor; ARMS-qPCR, amplification refractory mutation system-based quantitative polymerase chain reaction; ddPCR, droplet digital polymerase chain reaction.

Wild-type and *EGFR* T790M mutation plasmids were constructed and validated by sequencing. The plasmids were then quantified by ARMS-qPCR via the standard curve method using specific contents of HT29 gDNA. Copies of DNA (n=6–300) from the *EGFR* T790M mutation plasmid were spiked into 6,000 copies of wild-type *EGFR* plasmid to obtain plasmid samples with 0.1–5% mutation rates. Among the plasmid samples with 5, 1, 0.5 and 0.1% T790M mutation rates, the ARMS-qPCR technology stably identified mutation in the plasmid samples with 5 and 1% mutation rates. The ddPCR method reliably detected mutation in the plasmid samples with all four mutation rates and calculated that the exact copy numbers (mean values) of the samples were 398, 57, 24 and 6, respectively; no mutation-positive copies were detected in the gDNA of HT-29 cells. These results are presented in Table II and Fig. 1.

### EGFR mutation detection of the 10 FFPE tumor samples

**Figure 2:**
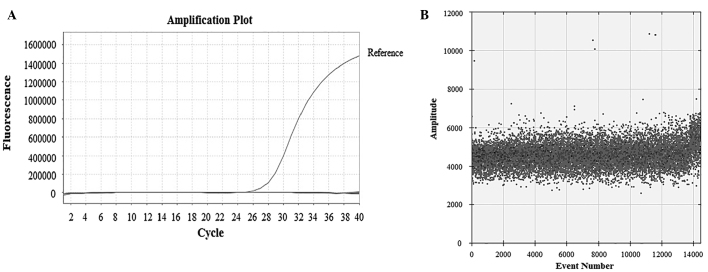
*EGFR* mutation detection results of the sample N006 by ARMS-qPCR and ddPCR methods. (A) The N006 sample was determined to be wild-type *EGFR* by qPCR. (B) The N006 sample showed the presence of seven mutated T790M alleles (0.1% mutant in the total genomic DNA from the tumor sample). EGFR, epidermal growth factor receptor; ARMS-qPCR, amplification refractory mutation system-based quantitative polymerase chain reaction; ddPCR, droplet digital polymerase chain reaction.

Of the 10 FFPE tumor samples, the detection results of nine samples were consistent in the ARMS-qPCR and ddPCR methods. In two (20%) of the 10 NSCLC tumor DNA samples tested, the presence of 84 and 153 molecules of an exon 19 deletion was identified among 6,000 genomic DNA copies (1.4% and 2.55%) in two samples of adenocarcinoma cell NSCLC tumor (stage IIA and IIB) obtained from a 68-year-old male and a 58-year-old female, respectively (Table III). In addition, the presence of L858R mutant *EGFR* alleles was identified in one (10%) of the 10 samples examined, which showed 164 mutated alleles (2.73%) among 6,000 genomic DNA copies (Table III). Notably, one sample named N006, which was considered as *EGFR* wild-type when analyzed by ARMS-qPCR, was demonstrated to be an *EGFR* T790M mutation with seven copies of the mutation being present. The comparison results of the two methods are presented in Table III, and the detailed results of sample N006 using the ARMS-qPCR and ddPCR approaches are shown in Fig. 2.

## Discussion

In this study, a series of plasmid samples with fractional *EGFR* T790M mutation rates, varying from 5 to 0.1% in a background of 6,000 wild-type copies, were examined by conventional ARMS-qPCR and the newer platform ddPCR. It was found that plasmid samples with mutation rates as low as 1% were stably detected by ARMS-qPCR, while plasmid samples with mutation rates from 5 to 0.1% were reliably detected by ddPCR, with high precision and reproducibility, and the exact copy numbers of the mutant sequences were measured simultaneously. Subsequently, 10 FFPE samples were used to explore the performances of ARMS-qPCR and ddPCR in the detection of *EGFR* mutation. Of the 10 FFPE samples, detection results for nine samples were totally consistent by the two methods, while one sample was determined to be wild-type *EGFR* by ARMS-qPCR but indicated to have an *EGFR* T790M mutation with seven copies of mutant molecules in a 6,000-copy wild-type background. These results illustrate that ddPCR can detect and quantify the presence of rare erlotinib/gefitinib-sensitizing *EGFR* mutations (0.12–2.73%) at a much higher sensitivity than is possible with conventional ARMS-qPCR.

In the comparison of ARMS-qPCR and ddPCR in clinical application, the interpretation of ARMS-qPCR results depends on the threshold and quantification cycle (Cq) value, and has a relatively high quality and concentration requirements for DNA templates (13). In this regard, ddPCR quantifies the sample by partitioning the sample and reaction components into thousands of reaction chambers, then counts the presence and absence of target molecules in each part of the sample following end-point PCR amplification (3,13); therefore, it is independent of the Cq value and has a lower requirement for the DNA sample. Furthermore, partitioning the sample could decrease the amount of background DNA in the partition, thus enhancing the mutant concentration in each reaction, giving greater target amplification specificity and sensitivity (3,13). Moreover, most clinical samples are FFPE tumor samples, in which DNA may be partially degraded (13); ddPCR is undoubtedly a good choice for these low quality DNA samples.

The results of the present study lend further support to the concept of *EGFR* mutation heterogeneity within lung tumors (14), in which *EGFR*-activating mutation exists to varying extents and even at very low abundances in early-stage lung tumors. Similar results were obtained in a earlier study involving the massively parallel sequencing of 22 lung adenocarcinoma specimens, which revealed that *EGFR* mutations can be very heterogeneous in a single tumor sample and that certain mutations were only present in <10% of total sequences (15). There is little literature concerning *EGFR* mutation detection by digital PCR. In 2009, Yung *et al* reported the feasibility of using digital arrays in the detection of *EGFR* mutations in tumor tissues from patients with advanced metastatic NSCLC at a detection limit of 0.1% of the total number of 10,000 *EGFR* sequences (13). However, the significance of detecting low abundance mutations in tumor tissues was unclear at that time (13). In a later study, the detection and quantification of rare gefitinib/erlotinib-sensitizing *EGFR* mutations (0.02–9.26% abundance) were reported in FFPE samples of early stage resectable lung tumors without an associated increase in gene copy number by digital array (3). The correlation between treatment response and low abundances of *EGFR* mutations was preliminary demonstrated in 2011; it was identified by direct DNA sequencing and ARMS approaches that lung cancer patients with low abundances (~1%) of *EGFR* mutations had a clearly longer median progression-free survival, higher objective response rate (ORR) and overall survival (OS) rate as compared with those of patient with wild-type *EGFR*, and the difference between patients with high and low abundances of *EGFR* mutations was not found to be significant regarding ORR and OS (16). Further research is required to clarify the significance of lower *EGFR* mutation abundance (<1%) detected by ddPCR.

In the two aforementioned papers concerning the detection of *EGFR* mutation by dPCR array, only in-frame deletion in exon 19 and L858R missense mutation in exon 21 were detected. Other gefitinib/erlotinib-sensitizing *EGFR* mutations, and more importantly, the T790M mutation, which can emerge during the treatment course with TKIs and then confer acquired drug resistance to the tumor cells, were not included. The *EGFR* mutant allele is heterogeneous, it may only present in a subset of tumor cells and can occur during cancer evolution; thus, the detection of the drug-resistant *EGFR* T790M mutation as early as possible before the acquired drug-resistance becomes clinically detectable is important, as it enables the physician to change the therapy promptly and give the patient the most effective treatment. In addition, ddPCR is a robust tool for achieving this objective, due to its high precision and sensitivity. Interestingly, the N006 sample in the present study, which was determined to be *EGFR* wild-type by ARMS-qPCR, was demonstrated to have an *EGFR* T790M mutation by ddPCR.

In summary, this study indicated the advantages of ddPCR in low abundance *EGFR* mutation detection, particularly the TKI-resistant associated *EGFR* T790M mutation. It is expected that ddPCR may have good application prospects in molecular cancer diagnosis, for example in the study of phosphoinositide-3-kinase, catalytic subunit α (PIK3CA) (17), MET proto-oncogene (MET; hepatocyte growth factor) (18) and Kirsten rat sarcoma viral oncogene homolog (K-RAS) (19). Since ddPCR is able to detect rare mutants in a high background of wild-type sequences, it may play an increasing role in the field of gene mutation in peripheral blood circulating DNA (20) and circulating tumor cells (21,22).

## Figures and Tables

**Table I:** Primers used for plasmid construction.

Primers	Direction	Sequences
Exon20-WT	Forward	CTTTTCCTCCATGAGTACG
Exon20-WT	Reverse	AACTCTTGCTATCCCAGG
T790M	Forward	ACCGTGCAGCTCATCATGCAGCTCATGC
T790M	Reverse	ATGATGAGCTGCACGGTGGAGGTGAGGC

WT, wild-type.

**Table II:** Detailed *EGFR* mutation detection results of the plasmid samples with 5–0.1% mutation abundance.

Target	Sample (%)	Copies	Positives	Negatives	Accepted droplets
T790M	5.0	398	245	13430	13675
T790M	1.0	57	35	13445	13480
T790M	0.5	24	14	12666	12680
T790M	0.5	24	15	13666	13681
T790M	0.1	7	4	13021	13025
T790M	0.1	5	3	13946	13949
T790M	HT-29	0	0	13608	13608

EGFR, epidermal growth factor receptor.

**Table III:** *EGFR* gene mutations in gDNA from samples of resected NSCLC tumors, as determined by the ARMS-qPCR and droplet digital PCR methods.

Sample name	Gender	Age, years	NSCLC histology subtype	Stage	ARMS-qPCR	ddPCR (copy numbers)	Consistency
N001	F	58	Adenocarcinoma	IIB	WT	WT	Yes
N002	M	47	Adenocarcinoma	IIIA	L858R	L858R (164)	Yes
N003	F	57	Squamous cell	IIA	WT	WT	Yes
N004	M	68	Adenocarcinoma	IIA	Del	Del (84)	Yes
N005	M	72	Adenocarcinoma	IIB	WT	WT	Yes
N006	F	75	Adenocarcinoma	IIIB	WT	T790M (7)	No
N007	F	58	Adenocarcinoma	IIB	Del	Del (153)	Yes
N008	M	73	Adenocarcinoma	IIB	WT	WT	Yes
N009	M	62	Squamous cell	IIIA	WT	WT	Yes
N010	F	54	Squamous cell	IA	WT	WT	Yes

EGFR, epidermal growth factor receptor; F, female; M, male; NSCLC, non-small cell lung cancer; ARMS-qPCR, amplification refractory mutation system-based quantitative polymerase chain reaction; ddPCR, droplet digital polymerase chain reaction; WT, wild-type; Del, deletion.

## Abbreviations

EGFR: epidermal growth factor receptor
NSCLC: non-small cell lung cancer
TKIs: tyrosine kinase inhibitors
ARMS-qPCR: amplification refractory mutation system-based quantitative polymerase chain reaction
ddPCR: droplet digital polymerase chain reaction
